# Contribution of protein phosphorylation to binding-induced folding of the SLBP–histone mRNA complex probed by phosphorus-31 NMR

**DOI:** 10.1016/j.fob.2014.10.002

**Published:** 2014-10-16

**Authors:** Roopa Thapar

**Affiliations:** Department of Biochemistry and Biophysics and Program in Molecular Biology and Biotechnology, University of North Carolina, Chapel Hill, NC 27599, USA

**Keywords:** IDP, intrinsically disordered protein, NMR, nuclear magnetic resonance, RBD, RNA-binding domain, SLBP, stem-loop binding protein, Stem-loop binding protein (SLBP), RNA-binding domain (RBD), Intrinsically disordered protein (IDP), Phosphorylation, Histone mRNA, Phosphorus-31 NMR

## Abstract

Phosphorus-31 (^31^P) NMR can be used to characterize the structure and dynamics of phosphorylated proteins. Here, I use ^31^P NMR to report on the chemical nature of a phosphothreonine that lies in the RNA binding domain of SLBP (stem-loop binding protein). SLBP is an intrinsically disordered protein and phosphorylation at this threonine promotes the assembly of the SLBP–RNA complex. The data show that the ^31^P chemical shift can be a good spectroscopic probe for phosphate-coupled folding and binding processes in intrinsically disordered proteins, particularly where the phosphate exhibits torsional strain and is involved in a network of hydrogen-bonding interactions.

## Highlights

• SLBP is an intrinsically disordered protein (IDP) in the absence of RNA.
• A phosphothreonine in the SLBP RNA-binding domain stabilizes the SLBP–histone mRNA complex.
• This phosphate exhibits torsional strain as revealed by its ^31^P NMR chemical shift.
• Phosphates can play structural roles in stabilizing tertiary structure in IDPs.
• ^31^P NMR can be a good spectroscopic probe for folding of phosphorylated IDPs.

## Graphical abstract

**Figure:**
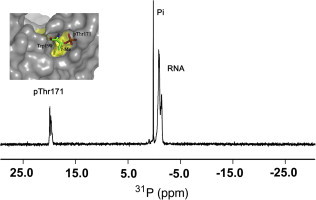

## 1 Introduction

Intrinsically disordered proteins (IDPs) are stably unfolded under physiological conditions and are highly prevalent in the eukaryotic genome [1,2]. Disordered regions in proteins can be sites of protein–protein or protein–nucleic acid interaction [3,4]. IDPs are known to undergo either disorder-to-order transitions [2,5] upon binding their partner, or they may form “fuzzy” complexes that remain dynamic in the complex [6–8]. Post-translational modifications or PTMs such as phosphorylation, acetylation, methylation, and ubiquitination also frequently occur in intrinsically disordered regions in proteins [9]. These PTMs can modulate the affinity of the IDP with its binding partner by either conformational selection [10] or induced fit mechanisms, or both [11]. Understanding how PTMs can modulate protein–protein or protein nucleic acid interactions in IDPs is important as it provides mechanistic understanding into how IDPs function in biological pathways.

Stem-loop binding protein (SLBP) is a histone mRNA specific RNA processing factor that forms a stable and specific complex with a 16 nucleotide stem-loop in the 3′ untranslated region of histone mRNA [12]. Human and *Drosophila* SLBPs are IDPs in the absence of RNA [13–16]. A unique feature of SLBP proteins is that they are phosphorylated at Thr171 (human SLBP numbering) in their RNA-binding domains (RBD) [17] and phosphorylation at this site is important for the kinetics of association with the RNA [17,15]. Dephosphorylation at Thr171 results in a ∼10-fold faster on rate and a ∼100-fold faster off-rate for the histone mRNA stem-loop [15]. The effect on the overall dissociation constant (*K_d_*) is ∼7–11-fold [15,17] lower affinity for the histone mRNA stem-loop, although there is a larger effect on the microscopic association (*k_on_*) and dissociation (*k_off_*) constants, and hence the kinetics of RNA binding [15]. NMR studies in solution also provide evidence for *cis*–*trans* isomerization about the Thr171–Pro172 sequence [15], and heteronuclear NMR studies show that mutation of Pro172 to glycine results in a single major conformation in solution [15]. Therefore the intrinsic disorder observed in solution is at least in part due to proline isomerization. Mutation of Pro172 also results in loss of RNA binding and embryonic lethality in *Caenorhabditis* *elegans* [15,18]. Consistent with this, the Pro172Gly mutant does not efficiently bind histone mRNA stem-loop in an EMSA assay [15]. In the crystal structures [19], the Pro172 ring is in the trans configuration, and shows van der Waals interactions with Trp183, Ile187, and Trp190. Mutation of Pro172 or isomerization to the cis-conformer would likely disrupt hydrophobic packing in this region and hence destabilize the SLBP RBD–RNA complex, as previously reported [15,20].

**Fig. 1:**
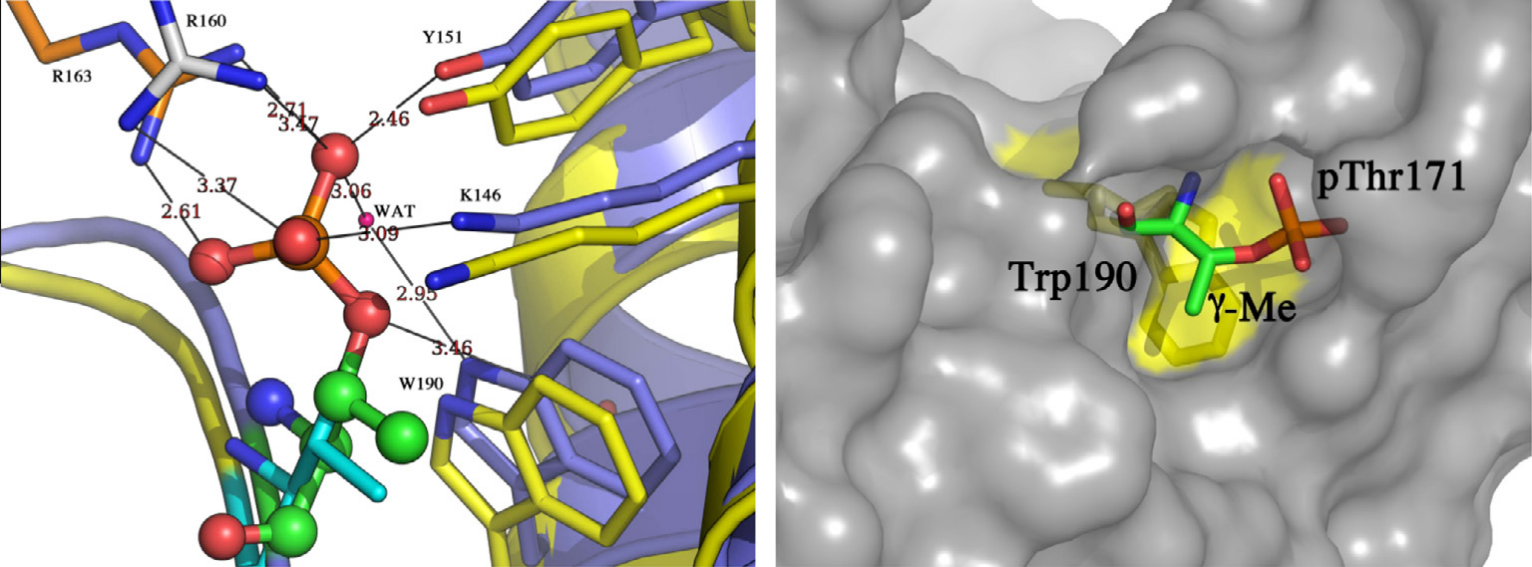
(Left) Electrostatic and aromatic environment around the phosphothreonine as observed in the crystal structure of the phosphorylated SLBP RBD–histone mRNA stem-loop-3′hExo ternary complex (PDB code 4QOZ) is shown in purple. The structure of the unphosphorylated SLBP RBD–histone mRNA stem-loop-3′hExo ternary complex (PDB code 4L8R) is superimposed in yellow. Hydrogen bonding interactions and distances to the phosphoryl oxygens (shown in ball and stick) are indicated. The γ-methyl group of the threonine is shown in green ball and stick. (Right) The surface of the SLBP RBD is shown. The phosphothreonine lies in a pocket where the phosphate group is solvent exposed while the γ-methyl group lies directly above the indole ring within van der Waals contact distance. (For interpretation of the references to color in this figure legend, the reader is referred to the web version of this article.)

Phosphorylation at Thr171 is important for subcellular localization of SLBP in the nucleus [20,21], for efficient histone mRNA processing [21] and mRNA decay [20], and SLBP protein stability *in vivo* [20] by regulating the stability of the SLBP–histone mRNA complex [22]. The structural environment around the phosphothreonine, as observed in the crystal structure of the human SLBP RBD complexed to histone mRNA stem-loop (Fig. 1), provides some insight into the role of threonine phosphorylation in RNA binding. When expressed in baculovirus, the 98-residue human SLBP RBD is phosphorylated at only one site (Thr171), as was previously confirmed by mass spectrometry [15,17]. In the crystal structure of the dephosphorylated SLBP–histone mRNA complex [19], no electron density is observed for 37 out of 99 residues in the SLBP RBD, particularly those surrounding the site of phosphorylation and the C-terminus. In addition, 57 out of 99 residues have high B-factors that may be attributed to the flexibility of the SLBP RBD. Phosphorylation of Thr171 stabilizes the structure of the RBD (PDB code 4QOZ), particularly around the phosphate. The phosphate is involved in a network of hydrogen-bonding interactions with residues in the three α-helices of the SLBP RBD in the presence of the RNA ligand, which helps stabilize the protein–RNA complex. The crystal structure is consistent with previous biophysical and NMR studies that showed that both phosphorylation and RNA binding of human [15] and *Drosophila* SLBP [13] proteins is important for stable recognition of the histone mRNA stem-loop.

Here I report that the ^31^P resonance for the phosphothreonine resonates 20 p.p.m downfield of H_3_PO_4_ in the SLBP–RNA complex. We previously reported this chemical shift in the ^31^P NMR spectrum of the baculovirus expressed *Drosophila* SLBP (dSLBP) RBD–histone mRNA stem-loop complex [13]. However, it was not possible to unambiguously attribute the shift to a single phosphate at the time since baculovirus-expressed dSLBP RBD is phosphorylated at the analogous threonine (T230 in dSLBP) [17] as well as four serines in the extreme C-terminus [23]. Here, ^31^P NMR has been used to describe the chemical nature of the phosphate corresponding to phosphorylated Thr171 and monitor the response of this phosphate in the human SLBP RBD to the presence of RNA. The ^31^P NMR data indicate that the orthophosphate that is covalently bonded to the threonine exhibits torsional strain in solution. The results have important implications for the role of phosphorylation in other IDPs. I propose that since many IDPs are phosphorylated, phosphates may play an important structural role in stabilizing the tertiary fold of such proteins, particularly in the presence of their ligands, and may also exhibit anomalous ^31^P chemical shifts as is observed for SLBP.

## 2 Materials and methods

### 2.1 Protein expression, purification, and NMR sample generation

A 128-residue pseudo-wild-type hSLBP RBD construct was designed to increase expression and ensure stoichiometric phosphorylation at Thr171. The hSLBP RBD (residues E118–E219) were cloned into the *Nco1* and *Xho1* restriction sites of the vector pFastBac™HTA (Invitrogen) and was expressed in Sf9 cells using the Bac-to-Bac expression system (Invitrogen) as previously described [15]. The protein was expressed in sf9 cells and purified using standard protocols used for Ni^2+^ affinity chromatography followed by gel filtration. Phosphorylation of the protein was confirmed by Electrospray Ionization Mass Spectrometry (ESI-MS) which gave a measured monoisotopic mass of 15255.40 Da corresponding to removal of the N-terminal Met (−131), acetylation of the new Ser (+42) N-terminus and phosphorylation of Thr171 (+80) as expected from previous studies [15,17]. Samples were concentrated and buffer exchanged using a G25 column into the NMR buffer (see below).

### 2.2 NMR spectroscopy

One-dimensional ^31^P NMR experiments were performed on a Varian Inova 500 MHz spectrometer using a broadband probe operating at a phosphorus frequency of 202 MHz. Unless otherwise noted, all measurements were made at 25 °C. For each experiment between 1000–20,000 transients were collected with a 65° excitation pulse, a recycle delay of 3 s, and a sweep width of 98.7 p.p.m with proton decoupling, unless otherwise noted. All ^31^P chemical shifts were referenced to 85% phosphoric acid. Experiments were recorded on 1–3 mM protein/peptide samples dissolved in 20 mM deuterated Tris, 50 mM NaCl, 0.1% sodium azide and 100% D_2_O. The hSLBP RBD–RNA complex samples contained a sixfold molar excess of RNA relative to protein.

## 3 Results and discussion

**Fig. 2:**
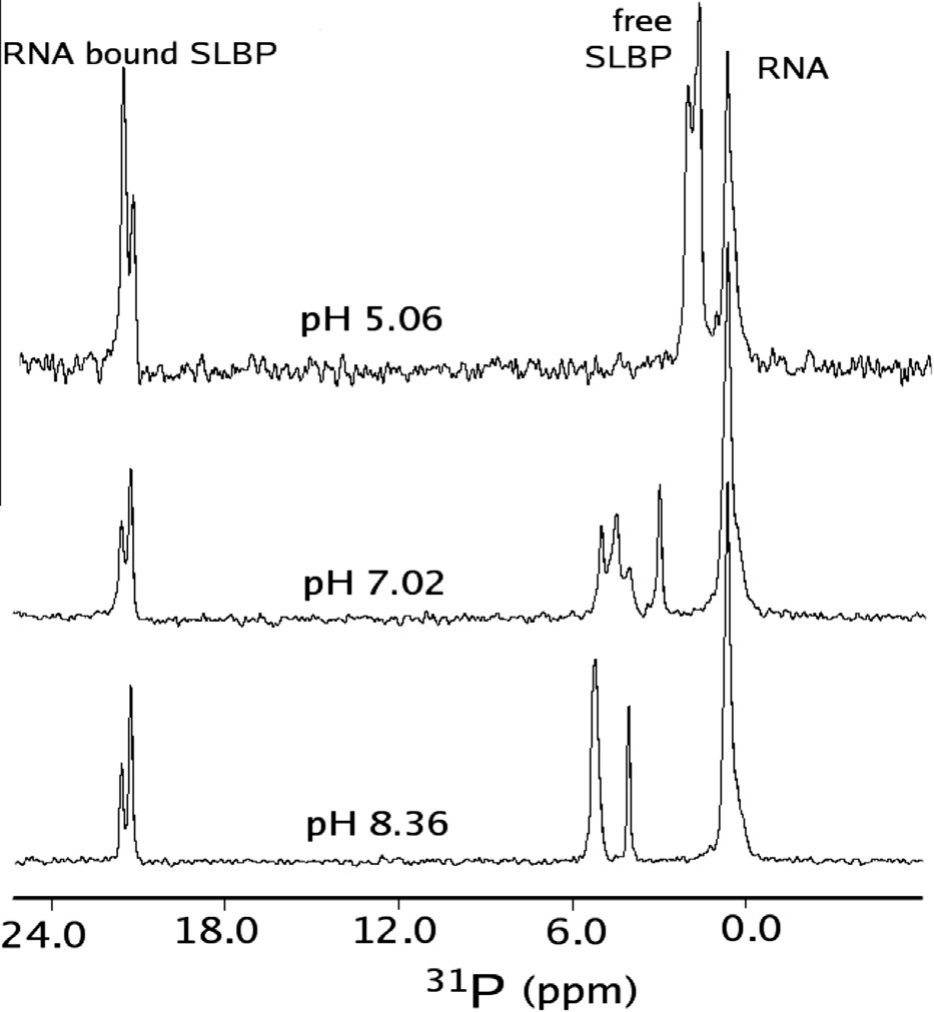
^31^P NMR spectra of the SLBP RBD–RNA complex collected at 25 °C at a spectrometer frequency of 202 MHz and at different pH values as indicated. A detailed pH titration is shown in Supplementary materials (Fig. S2).

**Fig. 3:**
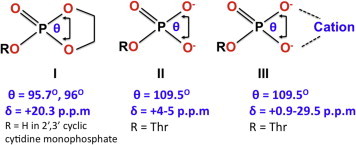
Expected ^31^P chemical shifts and dihedral angles due to the effect of structure and cations on an orthophosphate. The shifts are taken from references [25–28].

In the absence of RNA, two ^31^P NMR resonances are observed for a single phosphate in the hSLBP RBD (Fig. 2) at basic pH (pH > 8.5) at 3.00 p.p.m and 4.19 p.p.m, both of which lie within the range of that expected for o-phosphothreonine (3–5 p.p.m) [15]. The linewidths for these resonances are broad, consistent with the hypothesis that this domain undergoes conformational exchange between multiple states as previously reported for both *Drosophila* [13] and human [15] SLBP RBDs. In the absence of RNA, unphosphorylated and phosphorylated hSLBP and dSLBP RBDs are intrinsically disordered [13,15] with a large hydrodynamic radius that is characteristic of a pre-molten globule state [13]. At pH 8.1, the upfield-shifted resonance has an apparent linewidth of 28.2 Hz while the downfield resonance has an apparent linewidth of 89.5 Hz. This broad resonance comprises a number of conformational sub-states that are only slightly different in chemical shift at pH 8.1, but become more apparent at pH 7.0 (Fig. 2). When RNA is titrated into the free protein, the phosphothreonine resonance undergoes a remarkable downfield-shift (Fig. 2) to a resonance position (∼20 p.p.m downfield of 85% H_3_PO_4_) that may be attributed to a deviation of the O–P–O *σ*-bond angle from that of a perfect tetrahedron (109°28′) to that observed in a five-membered cyclic phosphate ester (I) [24] (Fig. 3) or an increase in chemical-shift anisotropy due to the electronegativity of the phosphate due to next-nearest-neighbor ligands as seen in cation–phosphate interactions [25]. It is not the chemical shift expected for a free dianionic orthophosphate (II) (Fig. 3). At least two conformations are observed in the RNA bound complex as well (at 20.02 and 20.3 p.p.m) suggesting that it remains dynamic when bound to RNA in solution.

To determine the ionization states of the phosphoryl groups, the ^31^P chemical shifts were followed as a function of pH in a pH range of 4–10 (Fig. 2, Supplementary Fig. S2). At neutral pH, the phosphoryl groups corresponding to free SLBP exist in multiple conformations in solution (Fig. 2) that are in slow exchange on the NMR timescale. Similar to inorganic phosphate (pKa 7.0 ± 0.01) and the o-phosphothreonine standard (pKa 6.3 ± 0.02), these ^31^P resonances go through a single ionization event corresponding to an apparent pKa of 6.9. The pH profile at pH values below 4.3 were not determined due to limited protein–RNA complex solubility under these conditions. Therefore, in the absence of RNA, and at neutral pH, the phosphothreonine in free SLBP favors a dianionic form that is dominant in all the observed conformational states. In contrast, the pH response of the two ^31^P resonances in the RNA bound form of the protein is flat and the chemical shift does not titrate with pH as would be expected for a dianionic phosphate inferred from the crystal structure (Fig. 2, Supplementary Fig. S2). The introduction of electrostatic interactions between a phosphoserine and a tyrosine and lysine in the KID–KIX complex has been reported to decrease the pKa of the phosphate to 4.3 ± 0.2 [26]. Therefore, it is not surprising that the network of electrostatic interactions mediated by the phosphate in the hSLBP RBD–RNA complex decreases the pKa of the phosphate to <4. Although the ^31^P resonances in the bound complexes do not exchange with pH, the relative populations of the two forms does change such that the downfield shifted conformer is preferred at acidic pH, whereas the upfield shifted conformer is preferred at basic pH.

**Fig. 4:**
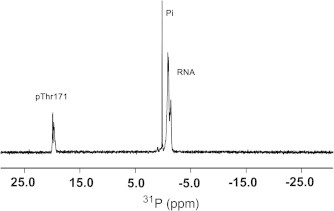
1D ^31^P NMR spectrum of the phosphorylated SLBP RBD–RNA complex collected after removal of free SLBP by gel filtration.

The unusual ^31^P shift observed in the SLBP–RNA complex is intriguing since such a downfield shift has never been previously reported for an orthophosphate. The only other protein for which an unusual ^31^P chemical shift has been reported is alkaline phosphatase where the catalytic phosphoserine was reported to resonate 8.5 p.p.m down field of H_3_PO_4_ and this effect was attributed to torsional strain [27,28]. No resonance is observed between +5 and +30 p.p.m in the spectrum of the free RNA or the NMR buffer (Supplementary Fig. S3). Temperature titration of the SLBP RBD in the presence of RNA (Supplementary Fig. S1) shows that the phosphate resonances of the free SLBP protein in solution exchange with those of the bound complex. No downfield shift is observed for a complex of non-phosphorylated SLBP and RNA (not shown). The addition of a denaturant such as urea to the hSLBP RBD–RNA complex results in a change in peak intensities for the resonances at 20 p.p.m and the free protein (Supplementary Fig. S4), as would be expected if the resonance came from the protein. ^31^P chemical shifts in RNA usually cluster between −3 and −1 p.p.m (upfield of orthophosphoric acid), however it is conceivable that unusual backbone torsional angles can shift a phosphate resonance originating from the RNA. The tetraloop structure of the RNA hairpin is significantly distorted and unfolded in the crystal structures of both phosphorylated and non-phosphorylated SLBP–RNA complexes [19,29]. To determine whether the shift can be attributed to the protein or the RNA, the SLBP RBD–RNA complex was purified over a gel filtration column to remove excess free SLBP RBD from the bound complex. A ^31^P NMR spectrum of the purified SLBP RBD complex shows only the downfield shifted resonance at 20.3 p.p.m and resonances between −3 and −1 p.p.m for the RNA (Fig. 4). No phosphate resonances are observed between 2 and 4 p.p.m for the phosphothreonine, as would be expected for a phosphothreonine in the free SLBP RBD. Taken together, the data shows that the anomalous ^31^P chemical shift in the SLBP–RNA complex can be directly attributed to the environment surrounding the site of phosphorylation and cannot be attributed to either the RNA or to a contamination in the protein/RNA complex.

Based upon quantum-mechanical calculations, Lechter and Van Wazer proposed 40 years ago that ^31^P chemical shift differences Δ*δ* arise from the sum of three contributing effects: the difference in the electronegativity of the P–X bond (Δ*χ*_x_) due to nearest-neighbor ligands, the change in the π-bonding orbitals about the phosphorus atom (Δ*n*_π_), and Δ*θ* or the change in the O–P–O σ-bond angle according to Eq. (1), where *C*, *k*, and *A* are constants,

(1) $$\Delta \delta =-C\Delta \chi_{\text{x}}+k\Delta n_{\pi }+A\Delta \theta$$

The depressed pKa of the phosphothreonine in the SLBP RBD–RNA complex suggests that these phosphates may exist in a strong electrostatic interaction with neighboring residues. In the crystal structure (PDB code 4QOZ), the phosphoryl oxygens are involved in a network of hydrogen bonds with Arg163, Tyr151, and Lys146 (Fig. 1). A weak hydrogen bonding interaction may also be present with Arg160 (the guanidinium ^η^N–O bond distances are 3.47 Å and 3.37 Å). A phosphoryl oxygen also makes a hydrogen bond to a water molecule, which is also coordinated to the Trp190 indole nitrogen. All known SLBPs have threonine conserved at position 171 (but never a serine) and tryptophan is also conserved at position 190 and is important for RNA binding [30]. The observed γ-methyl–π interaction likely contributes favorably to the SLBP–RNA complex stability, and also increasing the chemical-shift anisotropy on the phosphate, likely contributing to the downfield shift of the phosphorus-31 resonance.

No metal ion is observed in the vicinity of the phosphate in the crystal structure (PDB code 4QOZ). To test whether coordination of a metal ion to the phosphate in solution could explain this chemical shift behavior, up to 50 mM EDTA was titrated into the SLBP–RNA complex. Addition of EDTA had no appreciable change on the spectrum (data not shown). Addition of Mg^2+^ ions also did not have any appreciable effect on the downfield shifted peaks. Therefore perturbation of the π-electron cloud via metal-ion coordination does not explain the observed anomaly.

The contribution of electronegative effects to the ^31^P chemical shift is generally considered to be small [31]. A ∼2 p.p.m downfield shift (to 6 p.p.m) is reported for the KID–KIX complex where the phosphate participates in two hydrogen bonds [26]. Although the multitude of interactions mediated by the phosphoryl oxygens observed in the hSLBP RBD–RNA complex i.e. the change in the π-electron overlap (Δ*n*_π_) due to the hydrogen bonding interactions between the phosphoryl oxygens as well as the favorable γ-methyl interaction with the indole ring of Trp190 likely contributes to the large change in chemical shift observed for the phosphate resonance, it is unlikely to be the dominant or sole contributing factor.

The anomalous chemical shift observed in solution is most likely attributed to steric strain imposed by salt-bridging interactions as previously reported for alkaline phosphatase. Previous studies have shown that there is an empirical correlation between the O–P–O bond angle and the ^31^P chemical shift such that a decrease in the O–P–O bond angle by ∼3° is correlated with a downfield shift of the ^31^P resonance by ∼4 p.p.m [24]. The chemical shifts I report for the phosphate in SLBP are very close to those reported for five-membered cyclic phosphate esters in tetra co-ordinated phosphate compounds where the chemical shifts range between +10 and 20 p.p.m (Fig. 3(I)). The O–P–O bond angle surprisingly deviates from the ideal value of 109.5° and ranges between 89.49° and 127.77° over 74 PDB structures that have phosphothreonine for which the bond angles were measured (Supplementary Table 1) with several crystal structures showing decreased O–P–O bond angles. However, the measured O–P–O angles in the SLBP–RNA complex crystal structure are close to tetrahedral geometry (107.64°, 109.95°, 110.64°, and 109.92°). Contrary to this, the NMR data indicates that the phosphate in the hSLBP RBD exhibits torsional strain in solution, suggesting that the stereochemistry around the phosphate in solution may differ from that observed in the crystal.

## 4 Conclusions

The database of reported ^31^P chemical shifts for phosphorylated Ser/Thr/Tyr residues in proteins is small. The studies reported here along with previous studies on alkaline phosphatase suggest that orthophosphates can show anomalous chemical shifts in proteins due to the propensity of the phosphoryl oxygen to engage in a network of electrostatic interactions via nearest-neighbor effects on the oxygens as well as the geometry of the O–P–O bond. The presence of torsional strain on the phosphate may be particularly relevant in the case of intrinsically disordered proteins such as SLBP, where the phosphate brings together elements of secondary structure, thereby stabilizing the overall tertiary fold.

## Appendix A Supplementary data

Supplementary Table 1 and Figs. S1–4 This pdf file contains Supplementary Table S1 and Figs. S1–4.
