# Supplementary material for: Contribution of protein phosphorylation to binding-induced folding of the SLBP–histone mRNA complex probed by phosphorus-31 NMR
Source: FEBS Open Bio. 2014 Oct 16;4:853–7. doi: 10.1016/j.fob.2014.10.002 (PMC4215118; doi:10.1016/j.fob.2014.10.002)
Supplement: Supplementary Table 1 and Figs. S1–4 — This pdf file contains Supplementary Table S1 and Figs. S1–4. [file mmc1.pdf]

Supplementary Material for

Contribution of protein phosphorylation to binding induced folding of the SLBP-histone mRNA complex probed by phosphorus-31 NMR

Roopa Thapar

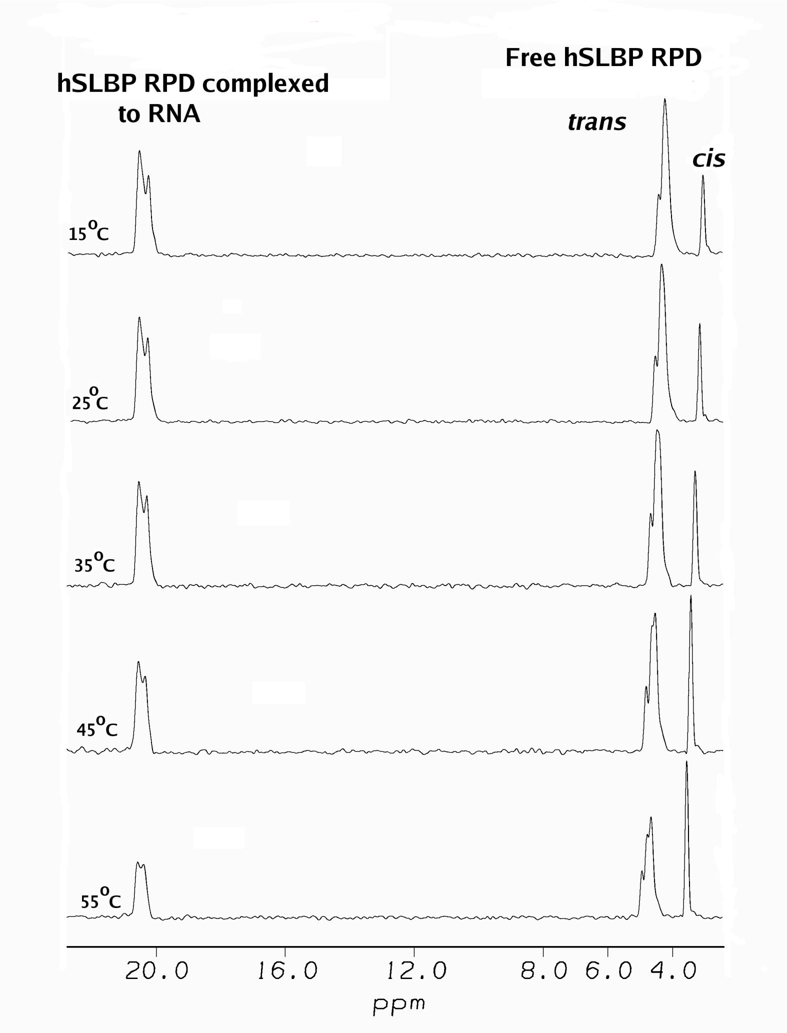

Figure S1. A temperature titration of the SLBP-RNA complex was performed between 15°C – 55°C on an Inova 500 MHz spectrometer as described under "Methods". Integration of NMR peak intensities and lineshape analysis confirms that resonances from the bound form (at ~20 p.p.m) exchange only with the upfield shifted resonances corresponding to free SLBP.

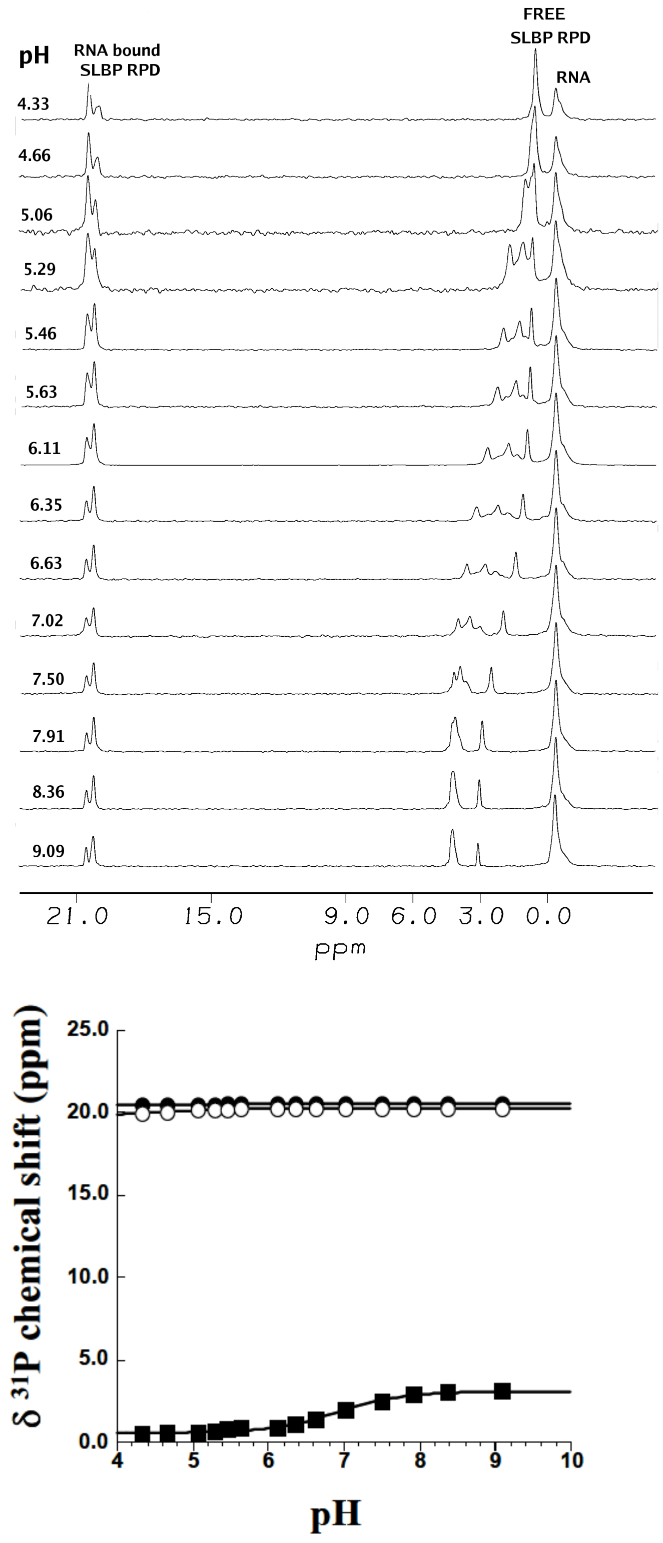

Figure S2. A pH titration of the SLBP-RNA complex shows the response of the ^{31}P nucleus in both the free and bound forms of SLBP to pH. The change in chemical shift was plotted vs. pH as shown above. The two peaks corresponding to the RNA-bound forms are shown in open and filled circles (●,○) whereas the titration of the upfield shifted resonance corresponding to free SLBP is shown in filled squares (■).

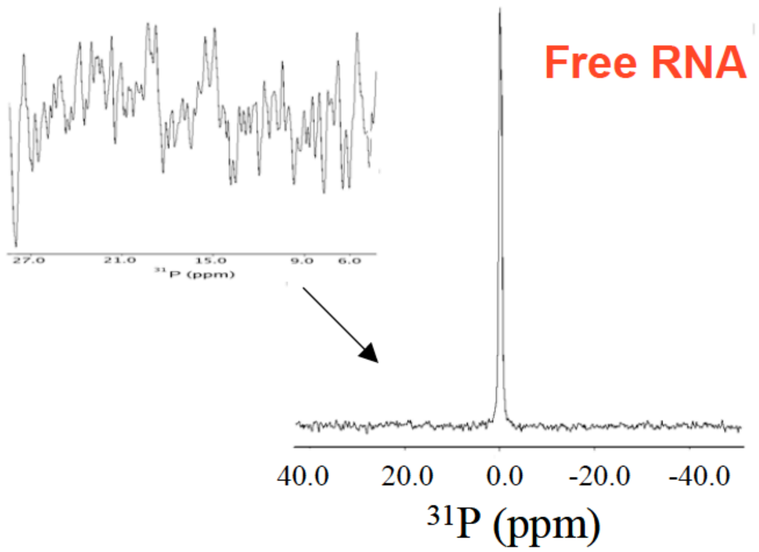

Figure S3. ^{31}P NMR spectrum of the free RNA collected with identical spectral parameters as for the protein-RNA complex. No resonance is observed at +20.3 p.p.m. indicating that the resonances we observe in the complex do not arise from a sample impurity in the RNA.

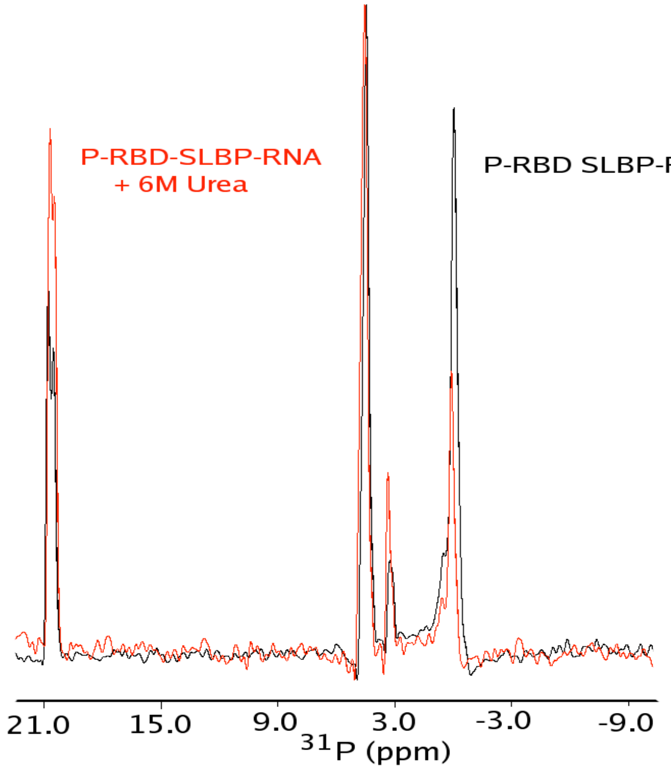

Figure S4. In black is shown the ^{31}P spectrum of the SLBP-RNA complex without urea. In red is shown the spectrum in the presence of 6 M urea. Changes in peak intensities are observed for the resonances in the complex, the free RNA, and free SLBP.

Supplementary Table 1. O-P-O bond angles measured for phosphothreonine in 74 crystal structures

**PDB Code**	**Structure**	**O-P-O ANGLES**
4QOZ	Crystal structure of the histone mRNA stem-loop, stem-loop binding protein (phosphorylated), and 3'hExo ternary complex	107.64, 109.95, 110.64, 109.92
4PSW	Crystal structure of histone acetyltransferase complex	113.15, 105.42, 105.59, 112.89
4UN0	Crystal structure of the human CDK12-cyclinK complex	109.27, 109.52, 109.50, 109.52
4KUJ	Structural and functional characterization of a novel Alpha Kinase from Entamoeba histolytica	104.71, 114.36, 114.62, 101.21
4O21	Product complex of metal-free PKAc, ATP-gamma-S and SP20.	110.14, 110.73, 108.30, 109.09
4CXA	Crystal structure of the human CDK12-cyclin K complex bound to AMPPNP	108.90, 109.37, 109.70, 109.71
4O0M	Crystal structure of T. Elongatus BP-1 Clock Protein KaiC	109.19, 109.34, 109.38, 109.22
4OGR	crystal structure of P-TEFb complex with AFF4 and Tat	108.60, 109.04, 110.14, 110.01
3WG7	A 1.9 angstrom radiation damage free X-ray structure of large (420KDa) protein by femtosecond crystallography	108.97, 106.74, 112.98, 105.47
4OR5	Crystal structure of HIV-1 Tat complexed with human P-TEFb and AFF4	111.73, 107.92, 106.86, 111.90
4BN1	Crystal structure of V174M mutant of Aurora-A kinase	107.20, 107.53, 113.91, 113.92
4JS8	Crystal structure of TTK kinase domain with an inhibitor: 401348	(phos #1) 109.49, 110.46, 107.96, 106.66 (phos #2) 108.80, 108.21, 109.75, 108.66
4NM5	Crystal structure of GSK-3/Axin complex bound to phosphorylated Wnt receptor LRP6 c-motif	108.84, 109.73, 109.81, 109.61
4NST	Crystal structure of human Cdk12/Cyclin K in complex with ADP-aluminum fluoride	105.72, 105.88, 115.27, 109.64
4CRS	Human Protein Kinase N2 (PKN2, PRKCL2) in complex with ATPgammaS	(phos #1) 103.57, 100.87, 118.01, 113.55 (phos #2) 105.92, 117.09, 103.64, 112.54
4C2V	Aurora B kinase in complex with the specific inhibitor Barasertib	109.58, 103.13, 115.75, 113.86
4C2W	Crystal structure of Aurora B in complex with	109.20, 100.31, 112.17,

	AMP-PNP	118.10
4OA2	Crystal structure of the BRI1 kinase domain (865-1196) in complex with ADP from Arabidopsis thaliana	102.34, 104.00, 115.38, 114.98
4OA9	Crystal structure of the BRI1 kinase domain (865-1160) in complex with AMPPNP and Mn from Arabidopsis thaliana	110.51, 101.23, 115.99, 112.19
4OAB	Crystal structure of the BRI1 kinase domain (865-1160) in complex with ATP from Arabidopsis thaliana	117.33, 102.61, 104.80, 110.78
4OAC	Crystal structure of the BRI1 kinase domain (865-1160) in complex with ADP from Arabidopsis thaliana	103.22, 113.01, 104.48, 110.59
4JDJ	Crystal structure of Serine/threonine-protein kinase PAK 4 F461V mutant in complex with Paktide T peptide substrate	114.41, 113.22, 104.49, 105.08
4JDK	Crystal structure of Serine/threonine-protein kinase PAK 4 F461V mutant in complex with Paktide S peptide substrate	101.03, 112.50, 112.85, 104.96
4CFE	Structure of full length human AMPK in complex with a small molecule activator, a benzimidazole derivative (991)	109.23, 109.36, 109.29, 109.38
4CFF	Structure of full length human AMPK in complex with a small molecule activator, a thienopyridone derivative (A-769662)	108.59, 109.59, 109.49, 109.60
4CFH	Structure of an active form of mammalian AMPK	109.54, 110.11, 110.06, 108.16
4CFN	Structure-based design of C8-substituted O6-cyclohexylmethoxyguanine CDK1 and 2 inhibitors.	105.37, 105.55, 104.98, 115.92
4CFW	Structure-based design of C8-substituted O6-cyclohexylmethoxyguanine CDK1 and 2 inhibitors.	115.81, 104.62, 115.00, 110.55
4IB0	X-ray Structure of cAMP dependent protein kinase A in complex with high Na+ concentration, ADP and phosphorylated peptide pSP20	109.14, 108.85, 107.25, 112.50
4IB1	Structure of cAMP dependent protein kinase A in complex with high K+ concentration, ADP and phosphorylated peptide pSP20	109.72, 110.05, 110.02, 109.48
4IB3	Structure of cAMP dependent protein kinase A in complex with ADP, phosphorylated peptide pSP20, and no metal	109.69, 109.35, 109.85, 109.25
4LKL	Crystal structure of Plk1 Polo-box domain in complex with PL-55	102.15, 101.36, 112.39, 113.88
4LKM	Crystal structure of Plk1 Polo-box domain in	114.17, 114.23, 103.58,

4LKM	Crystal structure of Plk1 Polo-box domain in complex with PL-74	114.17, 114.23, 103.58, 107.53
4C4E	Structure-based design of orally bioavailable pyrrolopyridine inhibitors of the mitotic kinase MPS1	112.56, 116.09, 109.32, 104.53
4C4F	Structure-based design of orally bioavailable pyrrolopyridine inhibitors of the mitotic kinase MPS1	113.99, 113.18, 107.12, 108.12
4C4G	Structure-based design of orally bioavailable pyrrolopyridine inhibitors of the mitotic kinase MPS1	108.77, 112.75, 113.42, 106.35
4C4J	Structure-based design of orally bioavailable pyrrolopyridine inhibitors of the mitotic kinase MPS1	105.99, 108.81, 112.99, 111.69
4LPA	Crystal structure of a Cdc6 phosphopeptide in complex with Cks1	109.34, 107.78, 107.90, 111.93
4N7T	Crystal structure of phosphorylated phosphopentomutase from streptococcus mutans	108.47, 109.97, 112.48, 107.62
4M69	Crystal structure of the mouse RIP3-MLKL complex	114.24, 104.46, 114.53, 103.77
4BU0	Crystal structure of Rad4 BRCT1,2 in complex with a Crb2 phosphopeptide	111.60, 118.92, 103.62, 112.68
4BU1	Crystal structure of Rad4 BRCT1,2 in complex with a Crb2 phosphopeptide	108.42, 108.26, 109.77, 110.61
4C33	PKA-S6K1 Chimera Apo	111.09, 103.53, 105.13, 111.26
4C34	PKA-S6K1 Chimera with Staurosporine bound	107.24, 113.09, 108.17, 107.89
4C35	PKA-S6K1 Chimera with compound 1 (NU1085) bound	109.78, 107.66, 109.82, 113.13
4C36	PKA-S6K1 Chimera with compound 15e (CCT147581) bound	117.23, 104.54, 108.31, 109.80
4C37	PKA-S6K1 Chimera with compound 21a (CCT196539) bound	108.65, 108.28, 115.96, 112.20
4C38	PKA-S6K1 Chimera with compound 21e (CCT239066) bound	108.22, 109.75, 113.22, 110.53
4L1U	Crystal Structure of Human Rtf1 Plus3 Domain in Complex with Spt5 CTR Phosphopeptide	109.75, 110.46, 108.68, 109.01
4BL0	Crystal structure of yeast Bub3-Bub1 bound to phospho-Spc105	109.20, 108.76, 108.77, 110.31
4EQC	Crystal structure of PAK1 kinase domain in complex with FRAX597 inhibitor	100.21, 117.12, 106.44, 112.68
4JG1	Structure of phosphoserine/threonine (pSTAb)	112.06, 107.42, 112.73,

4IMI	Novel Modifications on C-terminal Domain of RNA Polymerase II can Fine- tune the Phosphatase Activity of Ssu72.	114.73, 108.11, 104.07, 109.94
4LR7	Phosphopentomutase S154A variant	106.52, 101.82, 114.90, 113.91
4LR8	Phosphopentomutase S154A variant soaked with ribose 5-phosphate	114.22, 114.86, 107.70, 104.01
4LR9	Phosphopentomutase S154A variant soaked with 2,3-dideoxyribose 5-phosphate	107.57, 113.40, 114.23, 101.69
4LRA	Phosphopentomutase S154G variant	107.71, 102.85, 114.86, 101.36
4LRB	Phosphopentomutase S154G variant soaked with 2,3-dideoxyribose 5-phosphate	106.81, 102.42, 113.99, 114.64
4LRC	Phosphopentomutase V158L variant	100.74, 106.20, 106.63, 115.09
4LRD	Phosphopentomutase 4H11 variant	89.49, 125.64, 127.77, 91.58
4LRE	Phosphopentomutase soaked with 2,3-dideoxyribose 5-phosphate	113.86, 106.73, 103.81, 114.06
4LRF	Phosphopentomutase S154G variant soaked with ribose 5-phosphate	113.31, 114.85, 107.24, 102.12
4KAV	Crystal Structure of the soluble domain of Lipooligosaccharide phosphoethanolamine transferase A from Neisseria meningitidis	110.36, 107.69, 110.15, 116.30
4L46	Crystal structures of human p70S6K1-WT	111.79, 107.01, 107.37, 111.05
4KAY	Structure of the soluble domain of Lipooligosaccharide phosphoethanolamine transferase A from Neisseria meningitidis - complex with Zn	110.85, 107.44, 114.70, 110.99
4B8M	Aurora B kinase in complex with VX-680	113.87, 104.21, 102.45, 113.92
3UEO	Crystal structure of TopBP1 BRCT4/5 domains in complex with a phospho-peptide	108.75, 109.02, 106.80, 109.28
4IAC	X-RAY structure of cAMP dependent protein kinase A in compelx with HIGH MG2+ concentration, AMP-PCP AND pseudo-substrate peptide SP20	109.70, 109.54, 109.69, 109.28
4IAD	Low temperature X-ray Structure OF cAMP dependent protein kinase A in complex with high Mg2+ concentration, ADP and phosphorylated peptide pSP20	111.64, 108.47, 111.65, 108.06
4IAF	Room temperature X-ray Structure OF cAMP dependent protein kinase A in complex with high Mg2+ concentration, ADP and	109.19, 109.73, 109.12, 109.71

4IAF	Room temperature X-ray Structure OF cAMP dependent protein kinase A in complex with high Mg2+ concentration, ADP and phosphorylated peptide pSP20	109.19, 109.73, 109.12, 109.71
4IAI	X-ray Structure of cAMP dependent protein kinase A in complex with high Ca2+ concentration, ADP and phosphorylated peptide pSP20	108.79, 109.76, 109.79, 107.80
4IAK	Low temperature X-ray structure of cAMP dependent protein kinase A in complex with high Sr2+ concentration, ADP and phosphorylated peptide pSP20	109.91, 111.02, 110.09, 107.16
4IAY	Room temperature X-ray Structure of cAMP dependent protein kinase A in complex with high Sr2+ concentration, ADP and phosphorylated peptide pSP20	110.08, 110.03, 108.58, 108.93
